# Surface Modification of Zr_48_Cu_36_Al_8_Ag_8_ Bulk Metallic Glass through Glow Discharge Plasma Nitriding

**DOI:** 10.3390/ma17122850

**Published:** 2024-06-11

**Authors:** Krzysztof Kulikowski, Piotr Błyskun, Tomasz Borowski, Tadeusz Kulik

**Affiliations:** Faculty of Materials Science and Engineering, Warsaw University of Technology, Wołoska 141, 02-507 Warsaw, Poland; krzysztof.kulikowski@pw.edu.pl (K.K.); tomasz.borowski@pw.edu.pl (T.B.); tadeusz.kulik@pw.edu.pl (T.K.)

**Keywords:** bulk metallic glasses, surface treatment, glow discharge plasma nitriding, Zr_48_Cu_36_Al_8_Ag_8_ alloy, nanohardness

## Abstract

Bulk metallic glasses are modern engineering materials with unique functional properties. Zr-based alloys are particularly attractive as they exhibit high glass forming ability as well as good mechanical properties. Due to their relatively high thermal stability, reaching as much as 400 °C, they can be surface-treated in low-temperature plasma to further improve their mechanical properties. The subject of this study was to determine the influence of the technological parameters of nitriding in low-temperature plasma on the structure and mechanical properties of Zr_48_Cu_36_Al_8_Ag_8_ bulk metallic glass. In the course of this study, the influence of the ion accelerating voltage on the structure and micromechanical properties of the bulk metallic glass was analyzed. The produced samples were characterized in terms of nanohardness, layer adhesion by using the scratch test, and wear resistance by using the ball-on-disc method. As a result of low-temperature plasma nitriding, a significant increase in the surface nanohardness of the Zr_48_Cu_36_Al_8_Ag_8_ bulk metallic glass was obtained. The produced layers exhibited high adhesion to the substrate and they improved the wear resistance of the glass. The present study indicates the possibility of modifying the surface properties of bulk metallic glasses by using diffusion processes in low-temperature plasma without substrate crystallization.

## 1. Introduction

Modern engineering materials face multiple challenges. They are expected to be either cheap and easy to produce or exhibit unique properties that would allow them to be applied where price is not a major factor. Metallic glasses are materials that undoubtedly belong to the second group. They require advanced vacuum production technology that allows for rapid quenching of the molten alloy, which makes their preparation neither cheap nor easy. Nevertheless, these materials exhibit such extraordinary properties that they are still attractive candidates in some niche applications.

Among many currently known metallic glasses, the Zr-based ones are especially interesting as they combine very high glass forming ability (GFA) with good mechanical properties [1]. They can be prepared as large diameter casts [2] and applied as unique structural materials [3]. On top of that, Zr-based bulk metallic glasses (BMGs) are also known for their good corrosion resistance [4] and low coefficient of friction in contact with various other materials [5]. This set of properties has led to their use in the construction of micro-gears or sporting goods, for example [6].

One of the known methods to further improve the performance of a product is surface engineering. Surface treatments are often used to increase surface hardness and wear and scratch resistance, and sometimes to reduce the coefficient of friction. One of the most common surface treatments is nitriding, which is particularly popular in the steel industry for the production of parts for combustion engines or other elements particularly exposed to surface wear [7]. These types of surface treatments are usually carried out at elevated temperatures; therefore, their use is inappropriate for processing metallic glasses whose temperature stability is relatively low. However, plasma-assisted methods allow nitriding to be performed at temperatures as low as 573 K (300 °C) and the Zr-based alloys exhibit such high GFA that they can be held and processed around this temperature for many hours [8].

The idea of nitriding Zr-based BMGs is rather new as there have not been many studies describing it so far. Huang et al. performed nitrogen plasma immersion ion implantation and successfully improved the resistance to bio-corrosion of a Zr-based BMG [9]. In a more recent study, Huang et al. used laser irradiation in the nitrogen atmosphere to introduce nitrogen to the Zr-based BMG sample [10]. The ZrN phase embedded in the amorphous matrix increased the nanohardness of the alloy from about 6 to above 8 GPa, which is a significant improvement.

It is clear that nitrogen has a strengthening effect on Zr alloys. Lee et al. studied the influence of nitrogen content on the microstructure and mechanical properties of Zr-Cu-Ni-Al-N thin films, reaching a nanohardness of 10 GPa, although a nitrogen content above 8 at.% resulted in significant GFA deterioration [11]. On the other hand, a small amount of added nitrogen can in fact improve the GFA of Zr-Cu-based alloys, as was shown by Liu et al. [12]. Moreover, the ZrN phase, when introduced in small amounts through arc melting into the entire volume of Zr-based BMGs, can significantly improve compressive strength as well as plastic strain through shear bands proliferation mechanism [13]. However, other studies prove that nitrogen amounts above 1 at.% may lead to mechanical brittleness of these alloys [14]. These considerations show that the use of nitrogen to strengthen amorphous Zr alloys is fully justified. However, there are no reports in the literature on plasma-assisted surface nitriding processes so far.

In this study, the authors carried out several plasma-assisted nitriding processes of the Zr_48_Cu_36_Al_8_Ag_8_ BMG using various parameters. The surface treatments were performed below glass transition temperature to maintain the amorphous structure of the substrate. Surface properties such as nanohardness, the coefficient of friction, wear resistance and scratch resistance were investigated to assess the nitriding process influence on these mechanical properties.

## 2. Materials and Methods

The Zr-Cu-Ag-Al system is well known for its excellent glass forming ability [2]. In particular, the Zr_48_Cu_36_Al_8_Ag_8_ alloy exhibits very high crystallization resistance [8], and thus, it was selected as a substrate material for these studies. Master alloy was prepared by arc melting in an argon atmosphere (5N) with a titanium getter. The melting of pure elements (Zr—6N, other elements—4N) was repeated three times to homogenize the elemental distribution in the ingot. After that, rods with a diameter of 8 mm were prepared using the injection copper mould casting technique. Then, the rod ingots were cut into 2 mm thick discs with a precise low-speed diamond saw and subsequently grinded and polished to a mirror finish.

**Figure 1:**
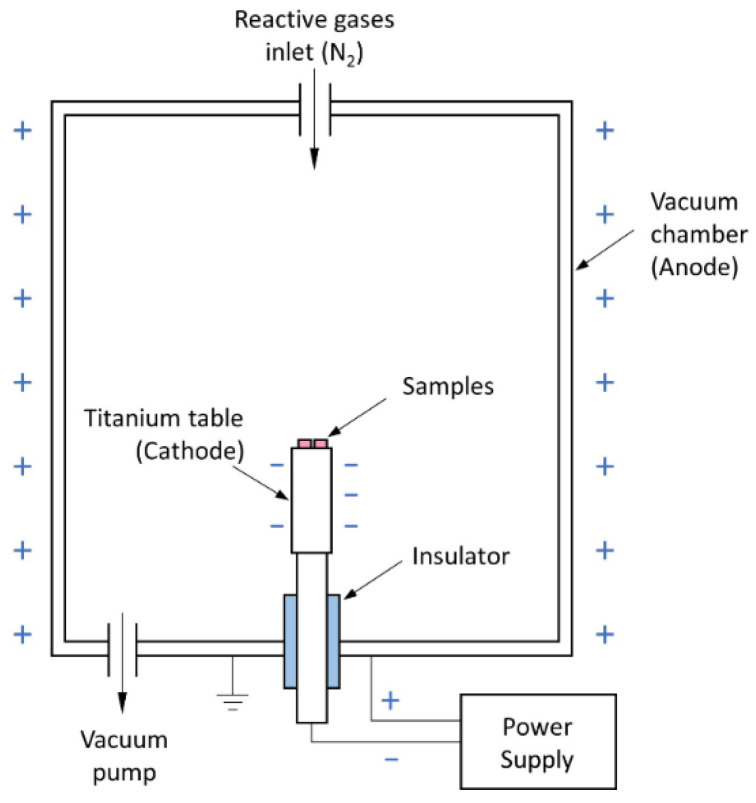
A diagram of the glow discharge nitriding device and an overview of the process.

Just before the nitriding process, the disc samples were cleaned ultrasonically in acetone to degrease the surface. Afterwards, they were placed on a titanium table inside a semi-industrial low-temperature plasma treatment apparatus (Figure 1).

In this method, samples on the table are separated from the chamber walls by the electrical insulator between the table and the walls. At the beginning of the process, the chamber is pumped down to a pressure of 10^−3^ mbar and then flushed with nitrogen at a pressure of 1 mbar to remove the residual oxygen. A reactive gas flows through the chamber during the process with a pressure in the range of 0.2–5 mbar. The table with the samples is polarized negatively in relation to the chamber walls. This polarization leads to the ionization of the reactive gas which is then accelerated towards the table for the reason of cathodic potential drop. Significant kinetic energy gained by the ions allows them to hit the treated surface where they are adsorbed or reacted with the substrate atoms. This relatively high kinetic energy of the ions allows for the occurrence of given chemical reactions at lower temperatures than in the case of non-ionized gas treatments. Technical nitrogen was used to carry out the surface nitriding processes of the metallic glassy substrate at 573 K (300 °C) for 30 min. These parameters are well within the thermodynamic stability range of this glass [8]. There were seven different ion accelerating voltage values applied: from 400 to 1000 V with a step of 100 V using the TruPlasma DC4030 power supply (Hüttinger Elektronik, Trumpf, Freiburg, Germany). During the process, a pulsed current with a frequency of 100 kHz was used. A higher voltage between the cathode and the anode increased the kinetic energy of the working gas ions. This in turn increased the probability of their adsorption or of a chemical reaction with the substrate.

X-ray diffraction phase analysis was performed on the Rigaku MiniFlex II diffractometer for the samples with a diameter of 8 mm after nitriding processes as well as for the non-treated sample for comparison. The following measurement conditions were used: CuKα radiation (λ = 1.54178 Å), 15 mA and 30 kV, and a step-scanning mode with a 3 s counting time and 0.05° step size. Diffraction angles 2θ ranged from 20° to 90°. The X-ray standard database ICDDPDF-4 + 2022 was used to support the phases’ determination.

Metallographic cross-sections of the treated discs were made to determine the thickness and quality of the produced nitride layers. The samples were embedded in a phenolic resin with graphite filler (for electron microscopy purposes) in such a way that the prepared layers were perpendicular to the section plane. The samples were then subjected to rough grinding using #80 grit paper to remove the first 2 mm of material to actually obtain the cross-section and not a side view. Then, this was followed by grinding on #240–1200 grit papers and polishing with a 1μm diamond suspension.

Cross-sections of the manufactured layers were observed on an AXIA ChemiSEM scanning electron microscope—SEM (Thermo Fisher Scientific Inc., Waltham, MA, USA)—equipped with an integrated EDS spectrometer, using secondary electrons (SE) and backscattered electrons (BSE) observation modes.

The NanoTest Vantage Alpha device from Micromaterials Ltd. (Wrexham, Wales, UK) was used to perform nanohardness measurements using a Berkovich indenter. The manufacturer software was used to correct the tip compression effect and equipment drift. The Oliver–Pharr method was used to calculate the nanohardness and indentation depth.

The measurements were carried out in two modes: using a constant and variable (multi-cycle) load. In order to determine the nanohardness of the structure formed during glow discharge nitriding processes, measurements under constant load were carried out using a load of 2 mN, for which the penetration depth did not exceed 120 nm. A total of 25 independent measurements were carried out for each layer, and then, the mean value and standard deviation were calculated. In turn, to determine the impact of surface layer formation on the nanohardness of the material, another study was carried out—a multi-cycle indentation study. A total of 20 load–unload cycles with loads from 0.5 to 500 mN were applied for each sample. Five independent measurements were performed for each sample. Due to the rather low reliability of the results obtained with a load of 0.5 mN, this measurement was used only as a locating tool. Thus, analysis of the nanohardness results at this stage was carried out beginning from the load of 39 mN. This approach provided information about the nanohardness values coming from various depths at the same spot. The low-load hardness values correspond to the surface zone of the samples, whereas the high-load values give information about the interactions between the layer and the substrate.

Adhesion of the produced nitride layers was analyzed with the Revetest (CSM Instruments, Peseux, Switzerland) scratch tester. A Rockwell diamond indenter of 0.2 mm in diameter was applied. On an ongoing basis, the pressure force, friction force, penetration depth and acoustic emission signal (layer cracking and decohesion) were recorded during the tests. The following parameters were employed: scratch length of 3 mm and normal force progressing from 1 N to 20 N. The light microscope Eclipse LV150N (Nikon, Tokyo, Japan) was used to perform microscopic observations of the scratches after the tests.

**Figure 2:**
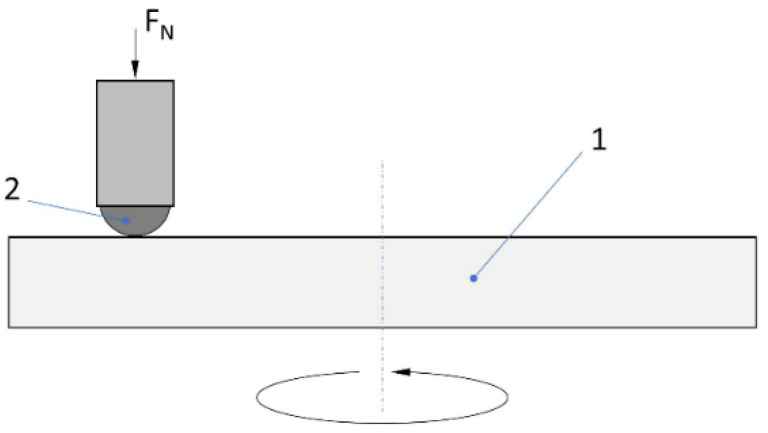
A schematic illustration of the “ball-on-disc” method. 1—sample (nitrided BMG); 2—countersample (Al_2_O_3_ ball).

A “ball-on-disc” method (T-21 tribotester by ITEE, Radom, Poland) was used to examine the wear resistance of the produced samples (Figure 2).

The tests were conducted dry (without lubrication) at a constant room temperature of 295 K (22 °C). A countersample of Al_2_O_3_ with a diameter of 6.35 mm was used. The tests were carried out under a load of 2 N, on a trace radius of 2 mm and with a linear speed of 0.1 m/s. A total of 1000 revolutions were performed, which is equivalent to a 12.56 m total distance travelled by the countersample.

## 3. Results and Discussion

### 3.1. Phase Composition Analysis

**Figure 3:**
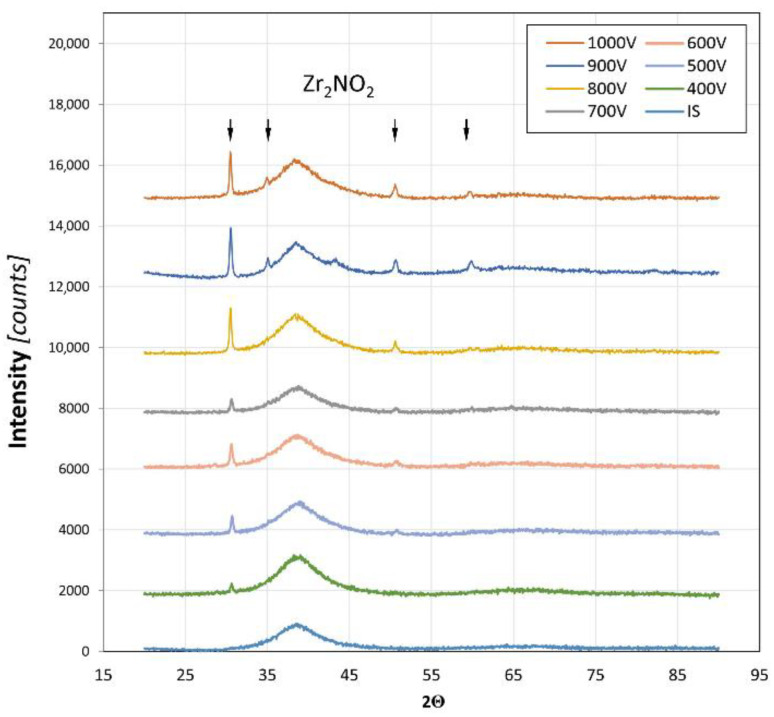
XRD phase analysis of the Zr_48_Cu_36_Al_8_Ag_8_ samples after the glow discharge nitriding process at an ion accelerating voltage ranging from 400 to 1000 V at 300 °C for 30 min.

The results of the XRD phase analysis are shown in Figure 3.

IS stands for the “initial state” sample of the Zr_48_Cu_36_Al_8_Ag_8_ glass without any surface treatments. There were no diffraction lines characteristic of crystalline phases present in the XRD curve for the IS sample, and thus, it is fully amorphous. However, for all other samples (ion accelerating voltage from 400 to 1000 V), at least one diffraction line was registered, proving that the nitriding processes resulted in a new phase formation in each case. All of these lines were attributed to the Zr_2_ON_2_ phase. The intensity of these lines increased with the ion accelerating voltage, which is related to the increase in the amount of this phase. This observation is consistent with the initial assumption that a higher voltage would lead to a more intense reaction of the working gas ions with the substrate material. It is also important to note that on each XRD curve, an amorphous halo is clearly visible. It shows that no substrate crystallization occurred during the surface treatments. Therefore, it is indeed possible to prepare a nitride layer on the surface of a BMG using glow discharge plasma nitriding while maintaining the substrate’s glassy structure.

The oxygen in the detected Zr_2_ON_2_ phase probably came from the samples’ surface pollution as the alloying components of Zr and Al are well known to exhibit high oxygen affinity and a thin oxide film is mostly always present on the surface of their alloys. Another source of oxygen pollution may be the reactive gas itself (nitrogen) as it was only of technical purity.

### 3.2. Nitride Layers Cross-Section Analysis

**Figure 4:**
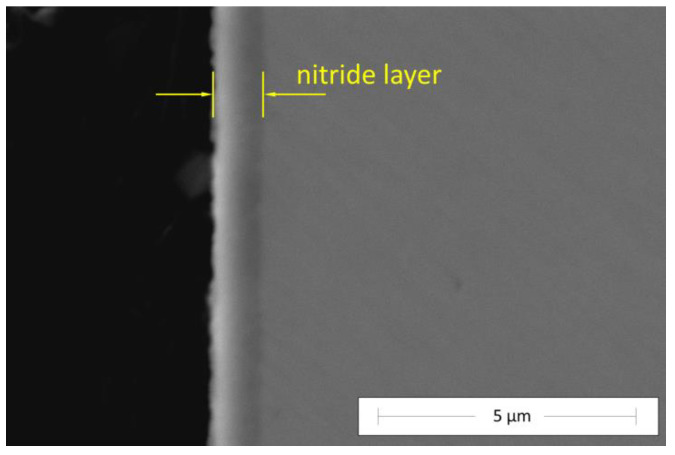
A SEM cross-section view of the nitride layer prepared on the Zr_48_Cu_36_Al_8_Ag_8_ glass at an ion accelerating voltage of 1000 V at 300 °C for 30 min.

An exemplary cross-section of the nitride layer prepared on the Zr_48_Cu_36_Al_8_Ag_8_ glass at an ion accelerating voltage of 1000 V at 300 °C for 30 min observed by using a SEM is presented in Figure 4.

The SEM studies indicated that all of the produced layers were fully homogenous. No separate areas could be distinguished inside them using this method. There were no visible defects or cracks, and thus, the layers’ adhesion to the substrate seemed to be promisingly good. Moreover, there were no signs of substrate crystallization, which is consistent with the XRD results (Figure 3) and is usually a major concern during surface treatments of such metastable materials.

### 3.3. Nanohardness

**Figure 5:**
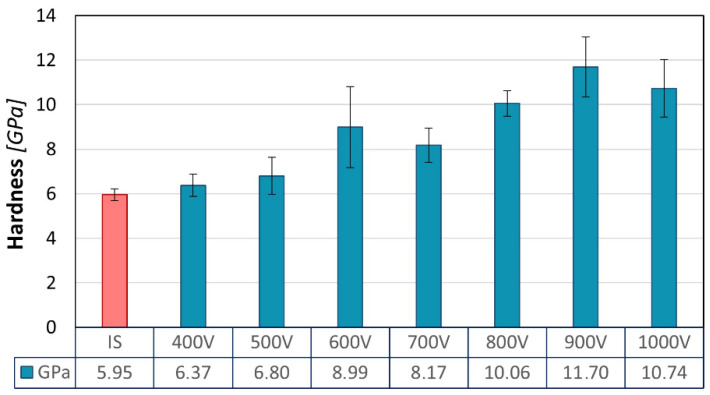
The ion accelerating voltage influence on the nanohardness of the nitrided samples, measured under a 2 mN constant load.

The Zr_48_Cu_36_Al_8_Ag_8_ glass in the initial state exhibited a nanohardness of approximately 5.95 ± 0.23 GPa under the 2 mN load (Figure 5).

As a result of the glow discharge nitriding processes, the surface nanohardness increased significantly along with the ion acceleration voltage. For low voltage samples (400–500 V), only a slight nanohardness increase was observed, not exceeding 15% of that measured for the non-treated glass (IS). At the same time, the measurement uncertainty increased accordingly. Higher process voltages increased the nanohardness, with a value of up to approximately 11.7 GPa for the sample nitrided at a voltage of 900 V. There were two slight deviations from this stable upward trend. The first one was observed in the case of the layer produced at the 600 V voltage, for which the nanohardness average value rose suddenly to about 8.6 GPa. The other contrasting deviation was measured for the sample nitrided at the 1000 V voltage, for which there was a decrease in the recorded nanohardness down to approx. 10.7 GPa compared to the nanohardness of 11.7 GPa for the layer produced at 900 V. However, it has to be noticed here that for both nitride layers produced at the highest voltages as well as for the 600 V voltage, nanohardness measurements were accompanied by a relatively large measurement error. If this error is taken into account, an increasing trend in nanohardness can easily be observed with increasing accelerating voltage.

**Figure 6:**
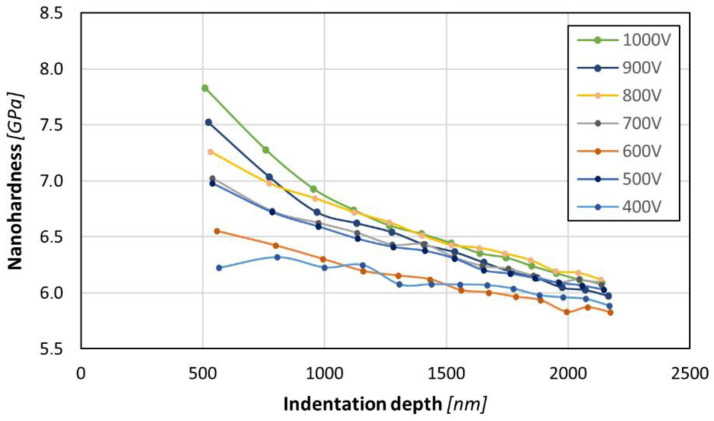
Nanohardness vs penetration depth for nitride layers prepared using ion accelerating voltage from 400 to 1000 V at 300 °C for 30 min, measured under 39–500 mN load.

Analysis of the nanohardness results with a cyclically varying load from 39 mN to 500 mN indicates a significant variation in the profile of nanohardness changes as a function of the indenter penetration depth (Figure 6).

The measurements carried out under a load of 39 mN show a similar tendency in terms of nanohardness vs ion accelerating voltage to the results obtained under a load of 2 mN (Figure 5), for which the penetration depth did not exceed 120 nm. A higher indenter load was accompanied by a larger area of stress influence on the material during the measurement, which allowed us to take into account the interactions between the layer and the substrate, which obviously had different properties. Analysis of the nanohardness under variable loads indicates that the highest values were achieved for the sample nitrided at 1000 V. The general correlation between the nanohardness and the ion accelerating voltage is observed in the entire range of applied voltages. Moreover, a significant decrease in the nanohardness is observed with an increase in the indenter load and penetration depth. Although this is generally a common trend in nanohardness measurements [15], this may indicate that a relatively high degree of strengthening occurred only in the top zone of the material, most likely to a depth not exceeding 1 μm. This observation corresponds well with the layer image in Figure 4. A higher penetration depth (at higher loads) resulted in lower nanohardness values in the case of all of the samples due to the increasing influence of the substrate with a lower nanohardness than the layer.

The nanohardness value registered for the IS sample (c.a. 6 GPa) is typical for Zr-based alloys [16]. The performed nitriding processes resulted in a significant increase in the nanohardness of all samples. The highest values registered with a load of 2 mN easily exceeded 10 GPa, which is a noteworthy improvement in the surface nanohardness in relation to the non-treated material. The nanohardness results obtained with higher loads (above 39 mN) ranged between 6 and 8 GPa. These values are very similar to those obtained by Huang et al. who nitrided a BMG surface using laser shock treatment [10].

### 3.4. Scratch Test

The scratch test method was used to test the adhesion of the prepared nitride layers and their resistance to mechanical failure up to an indenter load of 20 N. During the measurements, plastic deformation of the layer and the substrate was observed. The occurrence of cracks transverse to the wear trace was observed for all samples except for the layer produced at a voltage of 900 V, for which the cracks occurred only at the edge of the trace left by the indenter.

Table 1 shows the values of the critical load that led to the formation of cracks during the scratch test. Cracks near the wear trace usually appear during the scratch test [17].

**Figure 7:**
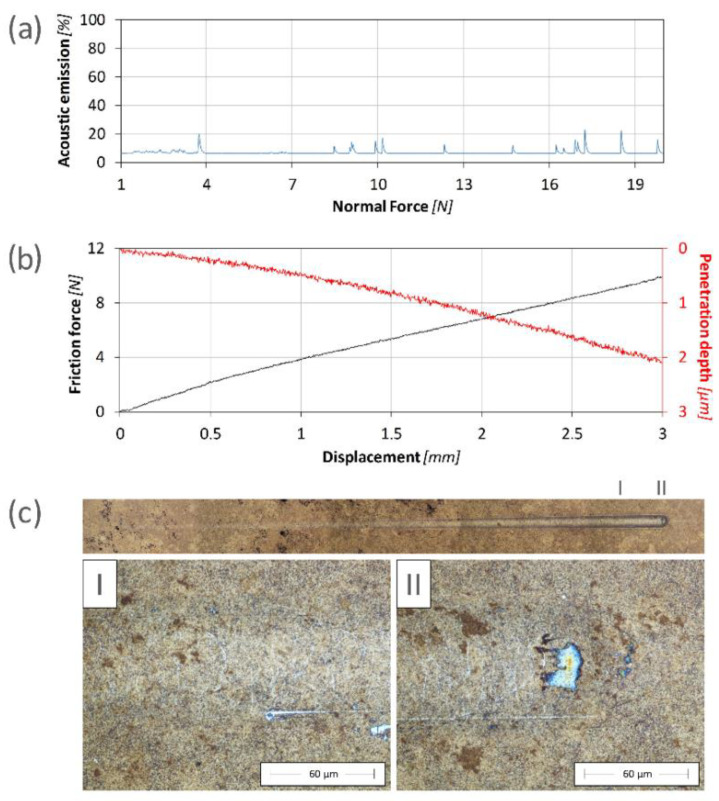
The results of the scratch test obtained for the sample nitrided at an ion accelerating voltage of 400 V: (**a**) the course of changes in acoustic emission vs load; (**b**) the friction and penetration depth vs indenter displacement; (**c**) scratch images in two different areas (I and II).

In the case of the layer produced at a voltage of 400 V, the occurrence of multiple cracks was observed at a load of approximately 17.1 N (Figure 7). Due to the very low thickness of the layer and its relatively low hardness, the appearance of cracks during the test did not result in a significant increase in the acoustic emission (Figure 7a). There were no abrupt changes in the friction coefficient or penetration depth (Figure 7b), which could indicate the penetration or delamination of the layer. This is confirmed through microscopic observations, during which no exposure of the substrate was observed up to the maximum test force of 20 N (Figure 7c).

The relatively high cracking resistance of the surface layer is most probably related to the rather low degree of strengthening of the material surface (a small difference in hardness between the produced layer and the substrate material). As a result, the distribution of stresses during the interaction of the substrate with the indenter is similar to the distribution occurring in homogeneous materials. Therefore, the modified surface zone is deformed evenly along with the substrate. However, a higher degree of material strengthening, which was the case for the higher ion acceleration voltages, changes the stress transfer characteristics. This is due to the decrease in the surface layer’s susceptibility to deformation along with the substrate. The impact of the indenter causes the strengthened surface layer to transfer stresses to the areas adjacent to the point of contact with the substrate, exceeding the cohesive forces of the layer, and thus leading to its premature cracking. For this reason, the critical load decreased with the ion accelerating voltage in the range of 400–800 V (Table 1).

**Figure 8:**
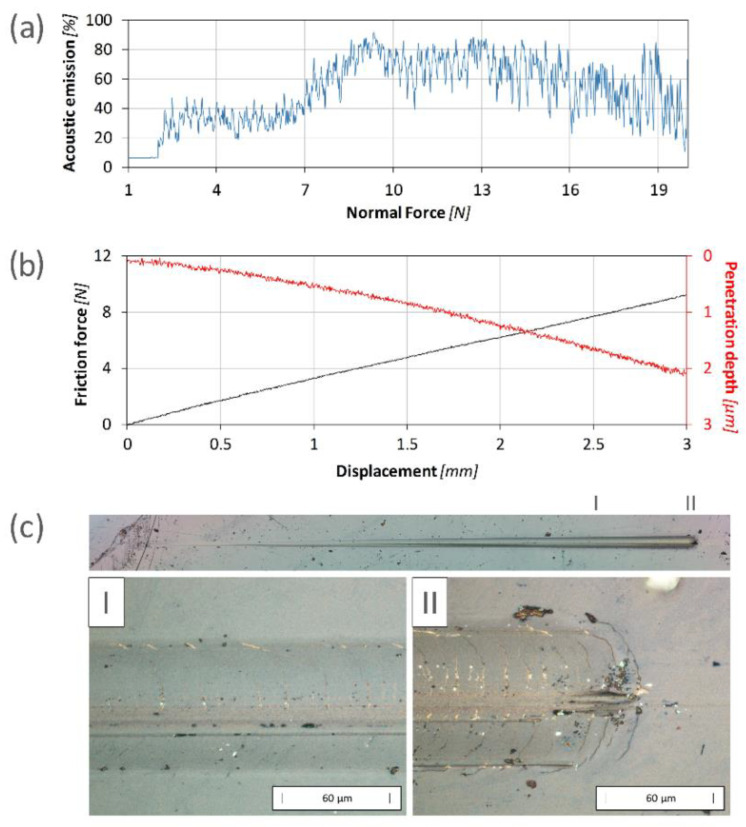
The results of the scratch test obtained for the sample nitrided with an ion accelerating voltage of 1000 V: (**a**) the course of changes in acoustic emission vs load; (**b**) the friction and penetration depth vs indenter displacement; (**c**) scratch images in two different areas (I and II).

On the other hand, the layers produced using voltages of 900 and 1000 V were characterized by a higher critical load L_c1_ (Table 1). The increase in thickness combined with a sudden increase in the hardness of the surface zone enabled the transfer of greater stresses into the layer plane (Figure 8). It should be noted here that the cracks that appeared in the early stage of the test were located at the side edges of the indenter trace. The higher hardness of the layer increased the stiffness of the surface zone. As a result, during the interaction of the indenter with the surface and the deformation of the substrate, the layer was sheared at the edges of the crack, causing a strong acoustic signal (Figure 8a). In the case of this sample, no abrupt changes in the friction coefficient or penetration depth were observed (Figure 8b), proving that neither penetration nor delamination of the layer occurred. The first transverse cracks (Figure 8c), related to the damage of the layer, were observed at loads of approximately 17.0 N (900 V) and 14.9 N (1000 V), respectively.

It is worth highlighting that no visible delamination or peeling of the substrate material, typical in scratch tests on these glasses [18], occurred in the current study. The only cracks were observed across the direction of the indenter movement, which resulted from the brittle fracture of the nitride layers during the deformation of the substrate. Despite that, at the bottom of the crack, there was no layer fragments displacement relative to the substrate. This means that the shear stresses resulting from friction in the movement direction did not exceed the stresses necessary to delaminate the surface layer in the entire load range (1–20 N). This is proof of good adhesion between the prepared layers and the glassy substrate.

### 3.5. Tribological Properties

**Figure 9:**
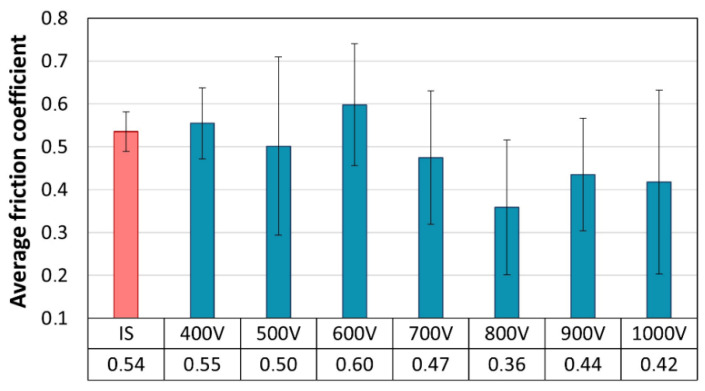
Average friction coefficient measured during ball-on-disc wear resistance test of samples nitrided at various ion accelerating voltages.

Figure 9 shows the average coefficient of friction values measured during the ball-on-disc test.

**Figure 10:**
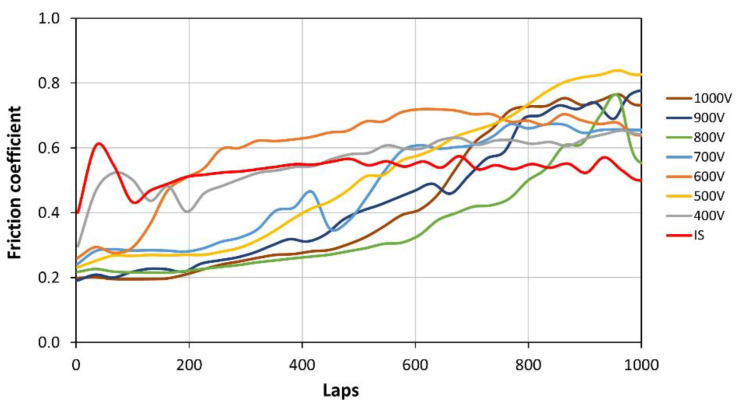
The course of changes in the friction coefficient during the ball-on-disc wear resistance test of the samples nitrided at various ion accelerating voltages.

**Figure 11:**
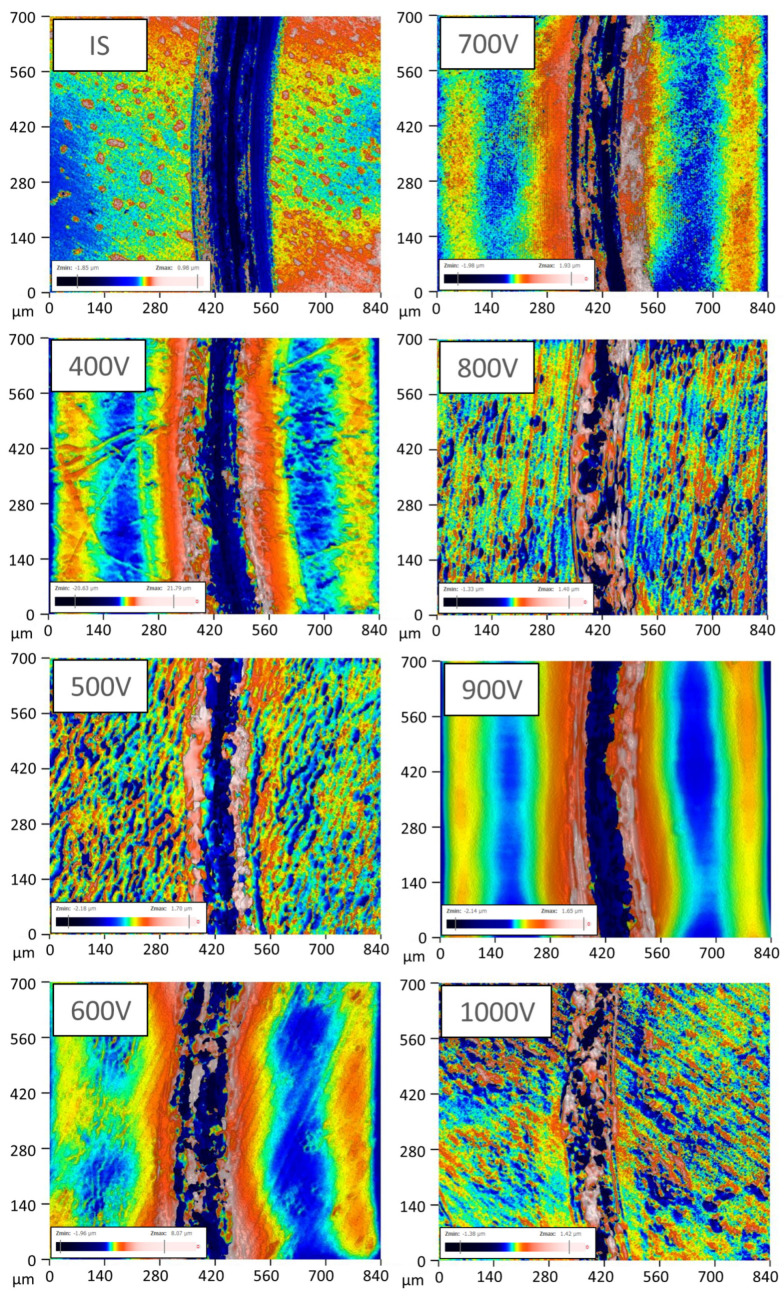
Surface topography of the wear traces from the ball-on-disc method performed on the initial state sample (IS) as well as on the samples nitrided at various ion accelerating voltages.

The course of changes in the friction coefficients is presented in Figure 10, whereas Figure 11 shows the profiles of wear traces.

The friction coefficient of the initial state sample in contact with the Al_2_O_3_ countersample stabilized during the test was at the level of approximately 0.54. The values of the average friction coefficients of the surface layers produced in the glow nitriding processes were quite similar (0.36–0.6). They did not differ much from the initial state. However, the values of the friction coefficients determined during the ball-disc test showed a significant scatter, presented as error bars in Figure 9. This resulted from the variability in the friction coefficient values during the measurement cycle due to the occurring material damage.

The initial values of the friction coefficients during the testing of the metallic glass with nitrided layers were much lower than in the case of the non-treated sample (IS) and ranged from 0.2 to 0.3 (Figure 10). These values correspond to the friction coefficient between the Al_2_O_3_ countersample and the nitrided surface layer [19]. As the number of friction measurement cycles increased, the surface layer of the material became damaged, leading to the exposure of the raw substrate. Possible interactions among hard wear products, the substrate and the countersample led to an increase in the friction coefficients up to values exceeding the coefficient recorded for the non-treated substrate. As a result, the recorded average friction coefficients have similar values (Figure 9), at a rather typical level for Zr-based glasses [20]. The course of changes in friction coefficients during the test indicates that materials with layers produced at a voltage of 800–1000 V retained a friction coefficient close to the initial one for the longest time. This indicates that they have the highest wear resistance and the highest resistance to mechanical failure of the surface layer. In the case of the remaining samples (400–700 V), the lower ion accelerating voltage was accompanied by an earlier increase in the friction coefficient, which indicates a lower degree of strengthening and potentially a lower thickness of the produced layer.

The wear traces presented in Figure 11 show that the mechanical damage that occurred during the ball-on-disc test was of abrasive nature. The wear trace on the initial state sample is rather clean, yet some wear products can be observed in wear traces on the nitrided samples. This debris could be responsible for the higher friction coefficient registered at the end of the tests for the nitrided samples in relation to the non-treated one (Figure 10). However, neither cracks nor chipping of the surface layer were detected. The largest wear trace was registered for the non-treated sample (IS). This observation corresponds well with previous results (Figure 9 and Figure 10). Moreover, it can be observed that the increase in the ion accelerating voltage resulted in a reduction in the wear trace. This is proof that glow discharge nitriding is a viable way of increasing the wear resistance of Zr-based metallic glasses.

The present study reveals that it is in fact possible to perform glow discharge nitriding processes in low-temperature plasma using the Zr_48_Cu_36_Al_8_Ag_8_ glass as a substrate. The process parameters of 300 °C and 30 min were sufficient to produce nitride layers using various ion accelerating voltages, yet the amorphous structure of the substrate did not undergo crystallization. The produced layers significantly improved the surface mechanical properties of the treated glass such as the nanohardness and wear resistance. It has been determined that the ion accelerating voltage is a parameter that can be used to effectively alter the nitride layers’ properties. Generally, the best properties were obtained for the highest used voltages. This novel approach to producing nitrided Zr-based BMGs allowed us to obtain very promising results of great importance as no such pioneering treatment has been reported in the literature so far. This is a good starting point to expand the scope of future research that would include other nitriding parameters studies, such as temperature and time of the treatment or maybe even combining the nitriding process with oxidation processes. The other interesting matter is to investigate the use of even higher voltages. However, the glow discharge treatment processes are carried out in the range of abnormal glow discharge, the maintenance of which requires correlation between the voltage, current and gas pressure in the working chamber. Standard processes have a voltage range limited to 1000 V, as above this level, the plasma often becomes unstable and enters the so-called discharge transition area, which causes arcing at higher currents. Theoretically, it is possible to implement such a treatment at higher voltages, but this would require a significant reduction in the gas pressure (below 0.1 mbar), which would additionally reduce the density of the ion stream flowing to the surface. To maintain process stability, it would be necessary to use a more precise pumping system and gas supply control, which may be a good idea for future research.

## 4. Conclusions

- The novel glow discharge nitriding processes allowed us to produce nitride layers on the surface of the Zr_48_Cu_36_Al_8_Ag_8_ bulk metallic glass without substrate crystallization.
- Only the Zr_2_ON_2_ phase was detected on the surface of every treated sample. The layers were microscopically fully homogenous and free of defects, such as cracks or delamination.
- The surface nanohardness increased with the ion accelerating voltage of nitriding. The highest nanohardness values were obtained for the samples nitrided with the highest voltages, which were almost twice as high as that of the non-treated glass.
- The scratch test results showed that the produced layers exhibited very good adhesion to the substrate and the critical force of cracking visibly depended on the nitriding process voltage.
- The nitride layers present on the glass surface reduced the initial friction coefficients significantly compared to the raw glass. The wear resistance of the nitrided samples was strongly influenced by the nitriding voltage.
- The best tribological properties were obtained for the samples nitrided with ion accelerating voltages of 900 and 1000 V.

## Figures and Tables

**Table 1:** The critical forces (L_c1_) that were registered during the scratch test of the samples nitrided at various ion accelerating voltages.

Ion Accelerating Voltage [V]	L_c1_ [N]
400	17.1
500	13.0
600	12.4
700	11.3
800	10.2
900	17.0
1000	14.9

## Data Availability

The raw data supporting the conclusions of this article will be made available by the authors on request.
